# Use of country of birth as an indicator of refugee background in health datasets

**DOI:** 10.1186/1471-2288-14-27

**Published:** 2014-02-19

**Authors:** Melanie Gibson-Helm, Jacqueline Boyle, Andrew Block, Helena Teede

**Affiliations:** 1 Monash Centre for Health Research and Implementation, School of Public Health and Preventive Medicine, Monash University, Locked Bag 29, Clayton, VIC 3168, Australia; 2 Refugee Health Service, Monash Health, David Street, Dandenong, VIC 3175, Australia; 3 Diabetes and Vascular Medicine Unit, Monash Health, Locked Bag 29, Clayton, VIC 3168, Australia

**Keywords:** Country of birth, Refugee, Data collection, Public health, Refugee health, Asylum seeker, Ethnicity, Migration

## Abstract

**Background:**

Routine public health databases contain a wealth of data useful for research among vulnerable or isolated groups, who may be under-represented in traditional medical research. Identifying specific vulnerable populations, such as resettled refugees, can be particularly challenging; often country of birth is the sole indicator of whether an individual has a refugee background. The objective of this article was to review strengths and weaknesses of different methodological approaches to identifying resettled refugees and comparison groups from routine health datasets and to propose the application of additional methodological rigour in future research.

**Discussion:**

Methodological approaches to selecting refugee and comparison groups from existing routine health datasets vary widely and are often explained in insufficient detail. Linked data systems or datasets from specialized refugee health services can accurately select resettled refugee and asylum seeker groups but have limited availability and can be selective. In contrast, country of birth is commonly collected in routine health datasets but a robust method for selecting humanitarian source countries based solely on this information is required. The authors recommend use of national immigration data to objectively identify countries of birth with high proportions of humanitarian entrants, matched by time period to the study dataset. When available, additional migration indicators may help to better understand migration as a health determinant. Methodologically, if multiple countries of birth are combined, the proportion of the sample represented by each country of birth should be included, with sub-analysis of individual countries of birth potentially providing further insights, if population size allows. United Nations-defined world regions provide an objective framework for combining countries of birth when necessary. A comparison group of economic migrants from the same world region may be appropriate if the resettlement country is particularly diverse ethnically or the refugee group differs in many ways to those born in the resettlement country.

**Summary:**

Routine health datasets are valuable resources for public health research; however rigorous methods for using country of birth to identify resettled refugees would optimize usefulness of these resources.

## Background

A wealth of data exists in routine hospital and primary care databases that may be valuable for research involving vulnerable or isolated populations, who for linguistic, cultural or societal reasons may under-represented in traditional medical research. These datasets have been created prior to generation of specific research questions and researchers face challenges concerning how to use these datasets most appropriately. One challenge is identifying specific vulnerable groups, such as those with a refugee background, using the available variables. Refugees may have poor health and specific health needs, however detailed studies are limited. Research has been hampered as many countries do not have linked immigration and health data systems and many health service databases do not collect residence permit information. Consequently, country of birth (COB) is often the sole indicator of whether an individual may have a refugee background. Year of arrival, indicating length of time in the resettlement country, is recommended for inclusion in routine health datasets, but has yet to be universally adopted [1,2]. Therefore a considered and robust method for using COB is required to identify probable resettled refugee and comparison groups accessing health services.

A refugee is someone who “owing to a well-founded fear of being persecuted for reasons of race, religion, nationality, membership of a particular social group or political opinion, is outside the country of his nationality, and is unable to, or owing to such fear, is unwilling to avail himself of the protection of that country” [3]. An asylum seeker is someone whose refugee claim has not yet been definitively evaluated [3]. Australia’s migration program consists of the Family Stream which seeks to reunite Australian residents with family members, the Skill Stream for those with abilities that will contribute to the Australian economy and the Humanitarian Program containing a number of visa types for refugees and asylum seekers [4]. An individual’s reason for migration is emerging as a possible contributor to health profiles and needs, hence the need for research among different migrant groups [5-7]. We aimed to design a study to investigate pregnancy outcomes among women of refugee background using an existing, routine hospital dataset and encountered significant barriers to defining women of refugee background. Key barriers included non-linkage of immigration and health data systems, no collection of immigration status or year of arrival and only COB was available. However, as one of the largest health service providers in Australia and with a substantial and diverse migrant population, the dataset had the potential to make a valuable contribution to our understanding of migration as a health determinant. Hence, the objective of this article is to discuss strengths and weaknesses of different methodological approaches to selecting resettled refugee and comparison groups from routine health datasets. Here, these methodological considerations are illustrated by examples from peer-reviewed literature and from a locally developed study to illustrate how some of the challenges can be addressed. This may assist readers to critically appraise literature from countries with different health systems to their own and assist researchers with both study design and manuscript preparation. While this article focuses on resettled refugees mainly within the context of maternal health, the issues raised are also applicable to general migration health research.

## Discussion

### Debate methods

The investigation of different methods for identifying refugee populations from routine datasets involved iterative searching of peer-reviewed literature. We specifically did not aim to conduct a systematic review as the topic did not lend itself to the Population, Intervention, Comparison, Outcome (PICO) framework. The initial search strategy was to identify articles describing maternal health and pregnancy outcomes among refugee populations. The articles were reviewed to ascertain whether an existing methodology could be applied to the proposed dataset. It emerged that the method depended on the available data and there was no consistent method for applying COB as a proxy for refugee background. Hence, the search strategy progressed to focus on articles that used routinely collected hospital/primary care databases to investigate health outcomes in general refugee populations, rather than solely focussing on pregnancy care, and in resettlement countries rather than transit countries or refugee camps. Consideration of strengths and weaknesses included whether the method accurately identified resettled refugees and its likelihood of selection bias, whether the method could be reproduced, and how the method may have affected results interpretation and comparison to other research. When COB was used as a proxy for refugee background, several key methodological questions emerged: how to define humanitarian source countries, what time period to use and whether to combine countries of birth. The learnings from this process then informed the methodology for a study using COB alone as a proxy for refugee background.

### Identification of resettled refugee groups

The method for selecting individuals with a refugee background depends on the data available to the researcher. This article focusses on three common methods: linked data systems, datasets from specialized refugee health services, and COB as the sole proxy for refugee background. The strengths and weaknesses of each method are discussed: accuracy of selecting refugee populations, method reproducibility and the method’s impact on results interpretation. A summary of this discussion is presented in Table 1.

**Table 1:** Methods used to select individuals of refugee background

**Population selection method**	**Examples of studies that used the method**	**Strengths of method**	**Weaknesses of method**
Linked data systems	Norredam, Garcia-Lopez, Keiding et al. 2009 [8]	• Uses a precise definition to accurately select individuals who have humanitarian residence permits.	• Use of the authority definition may misclassify individuals who have a refugee background but a non-humanitarian residence permit.
	Hollander, Bruce, Burstrom et al. 2011 [5]		
		• Can be used to select asylum seekers and/or refugees as separate groups.	
			• Not available in all countries or datasets so can be difficult to reproduce the method.
		• Facilitates simple results interpretation as the reader can be confident the sample is made up of individuals with a refugee background.	
			• May be difficulties comparing to countries that have different migration systems or authority definitions.
Datasets from specialized health services	Johnston, Smith & Roydhouse 2011 [10]	• Uses a precise definition to accurately select individuals who have humanitarian residence permits.	• Excludes individuals who have a refugee background but a non-humanitarian residence permit.
	Martin & Mak 2006 [11]	• Can be used to select asylum seekers and/or refugees as separate groups.	• Some to individuals of refugee background may not access specialized refugee health services, thus findings may not generalizable to whole refugee population.
		• Facilitates simple results interpretation as the reader can be confident the sample is made up of individuals with a refugee background.	
			• Residence permit type not commonly collected so can be difficult to reproduce the method using non-specialized datasets.
			• May be difficulties comparing to countries that have different migration systems or authority definitions.
COB alone as proxy for refugee background	Correa-Velez, Sundararajan, Brown et al. 2007 [19]	• Commonly collected by routine health datasets and therefore an easily reproducible method.	• Accuracy of selecting individuals of refugee background relies on an estimate of what proportion of individuals from each country of birth would be expected to be refugees.
	Correa-Velez & Ryan 2011 [24]		
		• Can be used to compare findings from countries that have different migration systems or authority definitions.	
			• Cannot be used to specifically select asylum seekers.
			• Not always enough information given to be confident the sample is primarily made up of individuals with a refugee background.

### Linked data systems

#### Strengths

A very precise method for selecting resettled refugees would involve direct cross-referencing of an individual’s health data against the same individual’s immigration data, therefore using the immigration authority’s exact definition of reason for migration [5,8]. For example, in Denmark the use of a unique identification number allows linkage between immigration data (migration status, type of residence permit, date of arrival) and health data [9], enabling accurate identification of groups of resettled refugees, family reunification migrants or asylum seekers. The precision of this method means the reader can be confident when interpreting results that the sample is comprised of individuals with a refugee background.

#### Weaknesses

Linked data systems are uncommon internationally and many researchers will be unable to reproduce this method. Additionally, using the authority definition may exclude those who have had refugee experiences but have a non-humanitarian residence permit. For example, Hollander et al. [5] selected individuals who were granted residence on refugee grounds and compared them to individuals from the same countries who were granted residence on family reunification grounds (as family members of refugees). Using the authority definition these family members were non-refugees, however they may also have had refugee experiences or considered themselves refugees [5]. Different countries have different migration systems and authority definitions, which may introduce difficulties in comparing results between countries.

### Datasets from specialized health services

#### Strengths

Refugee background may also be confirmed in some non-linked datasets from specialized refugee health services, where a humanitarian visa is a prerequisite for access to these services [10,11]. Likewise, some countries have refugee assistance programs which collect health outcome data specifically for asylum seekers [12,13]. As with linked data systems these datasets are likely to select refugee and asylum seeker groups accurately, using precise authority definitions that simplify results interpretation.

#### Weaknesses

In general, many health data collections do not include visa type, limiting reproducibility. Datasets from specialized refugee health services may also be limited by selection bias. Some individuals of refugee background may not access specialized refugee health services, with individuals accessing such services potentially not being representative of the wider resettled refugee population. These methods may also exclude individuals with a refugee background, but who have family reunification visas rather than humanitarian visas. Resettled refugee groups have sometimes been selected through specific pathology tests ordered as part of refugee post-arrival health assessments, followed by selection of specific ethnicities through reference to case notes [14]. However this approach again risks selection bias as some individuals may be missed, included in error or misclassified.

### COB alone as a proxy for refugee background

#### Strengths

When immigration and health systems are not linked or health data collections do not include visa type, COB is commonly used as a proxy measure of reason for migration. It is simple, fast and feasible to collect and therefore is commonly included in routine health datasets. It is comparable across datasets internationally, whereas immigration authority definitions differ between countries. It is an easily reproducible method, providing that enough detail is given about how it was used.

#### Weaknesses

COB is not a perfect indicator of refugee background; national immigration data can only provide an estimate of the likely proportion of refugees per COB in a given health dataset. There is not always enough information given to be confident the sample is primarily made up of individuals with a refugee background, making results interpretation and comparison difficult. COB alone also cannot differentiate asylum seekers from resettled refugees or other migrant groups. National immigration data and year of arrival may be used to estimate countries of birth with relatively high proportions of asylum seekers, but in some resettlement countries the absolute proportions may not be high enough to use COB alone as a proxy for asylum seeker status. Accurate selection of asylum seeker populations from existing routine datasets from mainstream health services would require information such as visa details.

#### Additional migration indicators

Migration status, residence permit type or COB cannot identify ethnicity, which requires further information such as language and religion [15]. Migration patterns can be complex and COB (or current migration status) may not accurately reflect where a person spent most of their time living [16]. To gain further insight into the complex relationship between migration and health, migration indicators additional to COB are recommended for routine collection in clinical health datasets: time since arrival in the country, language fluency, immigration status and other countries lived in (and length of time) [1,16]. Of these, year of arrival is likely to be the most feasible to add to current data collections [1].

#### Improving the use of COB as a proxy for refugee background

When using COB as a proxy for refugee background several decisions can be made to improve methodological rigor: how to define humanitarian source countries, what time period to use and whether to combine countries of birth.

#### Defining humanitarian source countries

Some articles assume that individuals from particular countries of birth are likely to have been refugees [17,18]. Supporting evidence for this assumption, such as inclusion of the proportion of immigrants from that COB who enter the resettlement country as refugees, is needed for method replication and for judging how accurately COB identified resettled refugees. Such evidence is also needed for confident results interpretation, assisting comparison to data from other resettlement countries with resettled refugees from that same COB or to compare to resettled refugees from other countries of birth. Correa-Velez et al. [19] provided a clear methodology using Australian immigration data to select countries of birth from which more than 80% of individuals entered Australia through the humanitarian migration stream [19]. This was a strong COB methodology as it included use of national immigration data to objectively identify countries of birth with high proportions of humanitarian entrants, matched by time period to the study dataset. Also, while the refugee group was analyzed as a whole, the proportion of the sample represented by each individual COB was included.

#### What time period to use

Changing migration patterns are also an important consideration for population selection, and whether the focus is short or long term effects of refugee experiences. Some studies focus on individuals from countries that have recently had a humanitarian crisis and assess the short-term effects of a refugee background on health outcomes [20]. Other studies focus on countries where humanitarian crises occurred some time ago and examine the interplay between long-term effects of refugee experiences and subsequent acculturation [21]. This is where routine collection of year of arrival in routine health datasets would assist in identification of probable refugee background and also would allow acculturation to be assessed more accurately [2].

#### Combining countries of birth

Often researchers combine humanitarian source countries into world regions for analysis [10,11,22]. Small population size or rare outcomes may sometimes necessitate this; however, when world regions are defined differently or different refugee groups are combined the findings can be difficult to compare or reproduce. For example, two studies investigated caesarean section rates in resettled refugee women but grouped the women differently [23,24]. The first was a study in Ireland that found no significant difference in caesarean sections for a combined refugee group, compared to the general hospital population [23]. The refugee group was predominantly African (specific countries of birth were not described) but also contained women from Romania, Kosovo, Russia and others. In Australia, a combined refugee group of only African-born women were reported less likely to have elective caesarean sections compared to all others birthing in the hospital [24]. Contrary to the results from both studies, a meta-analysis that included only Somali-born women reported significantly higher rates of caesarean sections compared to women born in the six receiving countries (Australia, Belgium, Canada, Finland, Norway and Sweden) [25]. It is difficult to compare these findings as the Irish study did not report which African countries the women were from and the Australian study did not report what proportion of Somali-born women in Australia at that time would be likely to have a refugee background. In another example of combining humanitarian source countries, two studies [10,11] investigated the health of confirmed refugees newly arrived to Australia; one applied Asia, Western and Central Africa, and Eastern Africa as the regions of birth [10], while the other applied South Asia, South East Asia, North Africa and sub-Saharan Africa and provided a list of countries included in each region [11]. If the reader was specifically interested in refugees from Burma, they couldn’t be sure whether the first study was relevant or not. The second study would have been strengthened by including the proportion of the sample represented by each individual COB.

Use of United Nations defined world regions has been suggested and provides a clear and reproducible framework for combining countries of birth [1,26] but no matter how world regions are defined, documentation of all countries represented in each world region is vital for comparison to other studies. Given the diversity of Africa and Asia, these general descriptors provide insufficient information without a breakdown of included countries. Tiong et al. [22] provided adequate information and combined African countries of birth into regions (Eastern, Western and Central Africa) and compared between regions, but also included the number of individuals from each country represented in the sample [22]. Other authors have reported results for one combined group but also have included the number of individuals from each country represented [24]. It is important to determine if specific health risks are common to all resettled refugees irrespective of COB or are increased only in particular world regions or countries of birth. Therefore, when population size allows, analysis of data at several levels (i.e. all individuals, then individuals from specific countries or regions) may be warranted. This is also where additional migration indicators, when available, could provide further insight into migration as a health determinant.

#### Choice of comparison group

The most common comparison group used in refugee health studies is one comprised of individuals born in the resettlement country. While this is valuable for highlighting differences between vulnerable groups and the general population, in many cases there will be substantial differences in culture, ethnicity, race and health behaviours between the two groups that may be difficult to quantify and include in analysis. Additionally, individuals born in a resettlement country may also comprise many diverse ethnicities. It is possible that comparing a combined heterogeneous group of individuals with refugee backgrounds to a combined heterogeneous group from a resettlement country leads to a compromised situation where results are not generalizable to any group of individuals. Along with different methods for selecting or defining the composition of the refugee sample, this may also contribute to difficulties interpreting and synthesising a body of literature [1]. Here more detailed comparisons to alternative groups may provide additional insights. For example, a comparison group may include countries of birth in the same or similar world regions, however the majority of immigrants may have been economic migrants. While Robertson et al. [27] compared resettled refugees to Swedish-born individuals, comparisons between economic migrants and the Swedish-born group were also included, providing some scope for interpreting refugee and non-refugee migrant results simultaneously [27]. Similarly, Janevic et al. [21] compared resettled refugees (former Yugoslavia) to individuals born in the resettlement country (United States of America) but also included a comparison between an economic migrant group from Poland and the American-born group [21]. Using overseas-born comparison groups is still an imperfect method as geographical boundaries can be somewhat arbitrary and may combine heterogeneous ethnic groups; however this last concern is also relevant almost any time COB is used, including for a comparison group made up of individuals born in a resettlement country [15].

#### Improving the use of COB as a proxy for refugee background: an example

Finally, the findings from the literature on COB were incorporated into the population selection method for a study investigating pregnancy outcomes among women of refugee background, compared to migrant women of non-refugee background. This study used an existing routine hospital dataset in Australia; the only indicator of refugee background was COB. National immigration data for the study period was used to select countries of birth where two thirds or more of the total immigrants had entered Australia within the humanitarian migration program (humanitarian source countries) and countries of birth where one third or less of the total immigrants had arrived within the humanitarian program (non-humanitarian source countries). This method allowed the reader to judge how accurately COB was likely to select women with a refugee background in this population and could be adapted or reproduced in countries that have different immigration systems. This method was also used to include women of contemporary refugee background but to exclude women from past humanitarian source countries from which contemporary migration was primarily non-refugee in nature. Women were selected from the hospital dataset if their COB was in the humanitarian source country category. From each United Nations-defined world region represented in the humanitarian source country group, all countries of birth in the non-humanitarian source country category were also selected from the hospital dataset as comparators. The study population consisted of 60 different countries of birth from six world regions: 14 humanitarian source countries and 46 non-humanitarian source countries. To determine if specific pregnancy outcomes were common to all resettled refugees or were increased only in particular world regions, analysis of the overall sample and then each world region was planned.

## Summary

Routine public health datasets provide an opportunity to investigate health care utilization and health outcomes among vulnerable groups of people, including those of refugee background. Linked data systems or datasets from specialized refugee health services use precise definitions to accurately select resettled refugee and asylum seeker groups but are not always available. COB is commonly collected in routine health datasets but a robust method for selecting humanitarian source countries is required. The authors recommend use of national immigration data to objectively identify countries of birth with high proportions of humanitarian entrants, matched by time period to the study dataset. If multiple countries of birth are combined, the proportion of the sample represented by each COB should be included. If population size allows, analysis of individual world regions or countries of birth may also be appropriate. United Nations-defined world regions provide an objective framework for combining countries of birth when necessary [26]. A comparison group of economic migrants from the same world region may provide additional insights. When available, additional migration indicators may also help to better understand migration as a health determinant.

A carefully developed and rigorous approach to use of COB when attempting to identify resettled refugee populations and full explanation of population selection in research articles would allow more meaningful comparison and synthesis of research results. This is needed to capitalize on available routine health data to improve health service provision and health outcomes among at-risk populations.

## Abbreviations

COB: Country of birth.

## Competing interests

The authors declare that they have no competing interests.

## Authors’ contributions

MGH contributed to conception and design, analysis and interpretation and drafted the manuscript. JB contributed to design, interpretation and revised the manuscript critically for important intellectual content. AB contributed to design, interpretation and revised the manuscript critically for important intellectual content. HT contributed to design, interpretation and revised the manuscript critically for important intellectual content. All authors read and approved the final manuscript.

## Pre-publication history

The pre-publication history for this paper can be accessed here:

http://www.biomedcentral.com/1471-2288/14/27/prepub
